# Application of Technology in Endocrine Disease

**DOI:** 10.1155/2014/715029

**Published:** 2014-02-12

**Authors:** Patrizio Tatti, Eldon D. Lehmann, Annabel E. Barber, Desiderio Passali, Felice Strollo

**Affiliations:** ^1^Department of Endocrinology and Diabetes, ASL RMH, Rome, Italy; ^2^Interventional Radiology Unit, North West London Hospitals NHS Trust (Northwick Park & St. Mark's Hospitals), Harrow, London HA1 3UJ, UK; ^3^General and Endocrine Surgery, University of Nevada School of Medicine, Las Vegas, NV, USA; ^4^ENT Clinic, University of Siena, Siena, Italy; ^5^Department of Metabolism, Nutrition and Wellness, INRCA-IRCCS, Rome, Italy

We live in a period of rapid change, in which many new technologies appear and are diffused through medical meetings, the Internet, specialized journals, the lay press, and media. While these innovations may bring a substantial improvement to the quality of care, sometimes, due to commercial pressures, technology can enter the market without adequate evidence of true benefit and without an analysis of the cost/benefit ratio. The converse is also true. Other potentially useful technologies can be underestimated due to insufficient commercial interest preventing further investment and development.

The present issue presents a selection of papers about the application of newer technologies in endocrinology. There have been numerous Special Issues devoted to the important topic of the application of information technology in clinical diabetes care [1–12], but to our best knowledge no attempts have been made at compiling a special issue regarding the application of technology in endocrinology.

The paper “*B-Flow twinkling sign in preoperative evaluation of cervical lymph nodes in patients with papillary thyroid carcinoma*” by G. Napolitano et al., from the Department of Health Science, University of Molise, Contrada Tappino, Campobasso, Italy, describes a new technique, B-flow imaging (BFI), to evaluate the presence of metastatic disease in lymph nodes. The authors report 99.7% specificity and 80.9% sensitivity for this technique which may be helpful in the preoperative planning of the intervention, prognostic staging, and individual therapy selection for patients.

The paper “*How to estimate fat mass in overweight and obese subjects*” by L. M. Donini et al., from the Medical Physiopathology Division, Food Science and Endocrinology Section, Food Science and Human Nutrition Research Unit, Experimental Medicine Department, Sapienza University of Rome, Italy, is of value to advance the understanding of the pathophysiology of obesity. This paper helps to make clear that body mass index (BMI), at the present state of our knowledge, is not more than a cursory evaluation, and not so useful to understand the pathophysiology and individualize the most appropriate treatment strategy. At the same time the report highlights the role of the other available techniques, including dual energy X-ray absorptiometry (DXA), body impedance analysis (BIA), and even the simple waist circumference (*W*) measurement. It is suggested that the widespread adoption of low-cost methods like BIA and *W*, in fact, could considerably help clinicians.

The paper “*Peripheral arterial tonometry to measure the effects of vardenafil on sympathetic tone in men with lifelong premature ejaculation*” by D. Francomano et al., from the Department of Experimental Medicine, Section of Medical Pathophysiology, Sapienza University of Rome, Italy, describes the use of peripheral arterial tonometry (PAT) to study the role of adrenergic overtone in men with lifelong premature ejaculation. This technique, coupled with intravaginal ejaculatory latency time (IELT), demonstrates a role of adrenergic hyperactivity, which can be reduced by the use of vardenafil on demand. This may be a helpful contribution to future research into this problem.

The remaining papers in the special issue focus on diabetes.

The paper “*Dynamic interactive educational diabetes simulations using the world wide web: an experience of more than 15 years with AIDA online*” by E. D. Lehmann et al., from Imperial College, University of London, UK, describes the development of a web-based version of the widely available downloadable http://www.2aida.org/ AIDA educational simulator of glucose-insulin interaction in diabetes. This utilizes a server-based architecture with HTML FORM commands to submit numerical data from a web-browser client to a remote web server. AIDA online, located on a remote server at http://www.2aida.net/, passes the received data through Perl scripts which interactively produce 24-hour insulin and glucose simulations. AIDA online allows users to modify the insulin regimen and diet of 40 different prestored “virtual diabetic patients,” on the Internet, or create new “patients” with user-generated regimens. Multiple simulations can be run, with graphical results viewed via a standard web-browser window. One of the relative strengths of AIDA online is that being totally Internet based; it can operate from any computer, anywhere, provided it has an Internet connection and a graphical display. This has led to widespread usage of the online simulator. To date, over 645,000 diabetes simulations have been run at AIDA online, from all over the world.

The remaining papers in this special issue deal with a nutritional algorithm developed by a group of experts that should gain wider acceptance. The algorithm supports the use of nutritional supplements in diabetes. This is a critical point because it is known that the diabetic population has subclinical or clinical malnutrition, correlated with the degree of hyperglycemia, the duration of the disease, and the medications used [13, 14].

This algorithm has a high degree of flexibility and the two papers in this issue show its applicability to different populations and dietary patterns. Transculturalization, as mentioned in the title, relies on the identification of appropriate, flexible guidance, which is available in the form of clinical practice guidelines (CPG). These guidelines, once reviewed for relevance and applicability by international experts, are abstracted and simplified in content and then condensed into a usable format. This process was applied to CPG in diabetes from the American Diabetes Association (ADA), the American Association of Clinical Endocrinologists (AACE), and other international organizations. The use of this technique should help dealing with an epidemic that could affect more that 300 million people in the world in the coming years, causing an unsustainable social and economic burden. The numbers are staggering. The world prevalence of diabetes is approximately 6.6% and in some countries exceeds 10%. Total financial liabilities related to the disease exceed US$370 billion globally [15].

Complicating matters are various cultural, clinical, and financial issues that prevent simple solutions and mandate an individual approach to patient care. Despite stemming from this same cultural and ideological root, the final two papers in this issue have different scope and focus.

The paper “*Transcultural diabetes nutrition algorithm: a Malaysian application*” by Z. Hussein et al., from the Department of Medicine, Hospital Putrajaya Pusat Pentadbiran Kerajaan Persekutuan, Putrajaya, Malaysia, describes a comprehensive management strategy to try and improve glycaemic control, which includes medical nutrition therapy (MNT). This report is mostly tailored to the needs of a population with a different standard of diabetes treatment and an Eastern diet, and may represent a paradigm for other populations with similar characteristics. The authors highlight that evidence-based recommendations for diabetes-specific therapeutic diets are available internationally. However, Asian patients with type 2 diabetes mellitus (T2D), including Malaysians, have unique disease characteristics and risk factors, as well as cultural and lifestyle dissimilarities, which may render international guidelines and recommendations less applicable and/or difficult to implement. With these thoughts in mind, a transcultural Diabetes Nutrition Algorithm (tDNA) was developed to account for cultural differences in lifestyle, diet, and genetic factors. The initial evidence-based global tDNA template was designed for simplicity, flexibility, and cultural modification. This paper reports the Malaysian adaptation of the tDNA, which takes into account the epidemiological, physiological, cultural, and lifestyle factors unique to Malaysia, as well as the local guidelines and recommendations.

The other paper dealing with diabetes in the special issue, “*The transcultural diabetes nutrition algorithm: a Canadian perspective*” by R. Gougeon et al., from Crabtree Nutrition Laboratories, McGill University Health Centre/Royal Victoria Hospital, Montreal, QC Canada, describes the Canadian version of the transcultural Diabetes Nutrition Algorithm which supports and targets behavioural changes to improve nutritional quality and to promote regular daily physical activity consistent with the Canadian Diabetes Association's CPG, as well as supporting the concomitant management of obesity, hypertension, dyslipidemia, and dysglycaemia in primary care. This report is evidently addressed to a Western population with high sanitary standard and deals mostly with the topic of the Glycemic Index.

It has not been possible to cover all relevant technologies in this special issue. As promising as all these reports are, all novel technologies must be evaluated, and beneficial effects scientifically proven. A future special issue may usefully address the formal validation and evaluation of such new technologies. Also diabetes/endocrine technology topics that could usefully be addressed include, but are not limited to the following.

*Endocrine*

- New technologies for the diagnosis of thyroid nodules (e.g., the role of 3D ultrasound).
- New technologies for the treatment of diabetic neuropathy (e.g., electrical stimulation for gastrointestinal paresis).
- New diagnostic, noninvasive techniques for neuroendocrine tumors.
- Evaluation of nanotechnologies.
- New technologies for the treatment of obesity.

*Diabetes*

- Evaluation of the technology for the treatment of diabetic foot ulcers.
- Diagnosis and treatment of obstructive sleep apnoea and impact on metabolic disorders.
- Meters with artificial intelligence for improved dosing recommendations and also artificial intelligence modelling, and educational and advisory software.
- Blood glucose meters, including invasive and noninvasive devices.
- Communication protocols and systems for implementing a robust and effective telediabetes infrastructure.
- New developments in continuous glucose monitoring with special focus on new sensor technologies.
- Insulin delivery systems, including pump technology.
- New tools and software for the “closure of the loop” in type 1 diabetes.
- Methods which combine a continuous BGL sensor, insulin pump, and control algorithms for creating an artificial pancreas.
- The role of applications (apps) for smart phones and handheld devices in ambulatory diabetes care.
- Diabetes management systems for transition from home to hospital and from hospital to home.
